# Planificar la muerte digna: un abordaje clínico y educativo internacional al amparo del conocimiento ciudadano

**DOI:** 10.15446/rsap.V25n5.109354

**Published:** 2023-09-01

**Authors:** José Manuel Jiménez-Rodríguez

**Affiliations:** 1 JJ: Lic. Antropología Social y Cultural. Ph. D. Ciencias Sociales, Facultad de Trabajo Social, Departamento de Sociología, Trabajo Social y Salud Pública, Universidad de Huelva, Campus "El Carmen". Huelva, España. jose.jimenez@dstso.uhu.es Universidad de Huelva Facultad de Trabajo Social Departamento de Sociología, Trabajo Social y Salud Pública Universidad de Huelva Huelva España jose.jimenez@dstso.uhu.es

**Keywords:** Planificación anticipada de las decisiones, muerte, conocimiento, percepción, educación *(fuente: DeCS, BIREME)*, Advance decision planning, death, knowledge, perception, education *(source: MeSH, NLM)*

## Abstract

**Objetivo:**

Identificar el conocimiento y la percepción del alumnado universitario sobre el proceso de la muerte digna.

**Métodos:**

Estudio descriptivo transversal en el que participan estudiantes de postgrado de la Universidad Internacional de la Rioja (España), matriculados durante el curso académico 2020-2021. Se lleva a cabo un muestreo aleatorio simple cuya N=215. Son criterios de inclusión, tener una edad entre 25 y 65 años y un claro dominio del castellano. Para la recolección de datos se hace uso del cuestionario autoadministrado. Se realiza un análisis descriptivo y bivariante de los datos obtenidos.

**Resultados:**

Participan 136 individuos. El conocimiento sobre el derecho a la muerte digna y su normativa representa una media de 5,43. El 6,56 considera que en su entorno se habla abiertamente sobre la muerte, y el 6,59 cree que en su ciudad la responsabilidad sobre el proceso de salud-enfermedad del paciente recae sobre el médico. Una media de 9,22 ve necesario que los colegios ofrezcan al alumnado información/formación sobre el proceso de la vida y la muerte.

**Discusión:**

Los participantes del estudio presentan un conocimiento justo sobre la regulación de la muerte digna, pero perciben favorablemente este derecho y consideran la necesidad de su divulgación desde las instituciones sanitarias y educativas.

## Abstract (en)

**Objective:**

To identify university students' knowledge and perception of the process of dignified death.

**Methods:**

Cross-sectional descriptive study involving postgraduate students of the International University of La Rioja (Spain), enrolled during the academic year 2020-2021. A simple random sampling was carried out with N=215. Inclusion criteria were to be aged between 25-65 years and have a clear command of Spanish. A self-adminis-tered questionnaire was used for data collection. A descriptive and bivariate analysis of the data obtained was carried out.

**Results:**

136 individuals participated. Knowledge of the right to a dignified death and its regulations represented an average of 5.43. The 6.56 think that in their environment peo-ple talk openly about death, and 6.59 believe that in their city the responsibility for the patient's health-illness process lies with the doctor. An average of 9.22 think it is necessary for schools to provide pupils with information/education on the process of life and death.

**Discussion:**

the participants in the study have a fair knowledge of the regulation of death with dignity, but they perceive this right favourably and consider the need for its dissemination by health and educational institutions.

Hablar con naturalidad sobre la muerte y todo aquello que la rodea sigue siendo un tema tabú, marcado por la superstición y la aprensión, entre otros factores. Se trata de un asunto que suele ser eludido y relegado a la esfera más íntima de la persona y su entorno inmediato. La falta de espontaneidad para el diálogo abierto y el abordaje de la muerte en consulta clínica dificulta la participación del paciente en su proceso de salud-enfermedad. Tal hecho favorece el obstinamiento terapéutico cuando el paciente se encuentra en situación mortecina 1,2.

El deseo de morir dignamente es un derecho sanitario reconocido en numerosos países de Europa y del mundo, recogido en la cartera de servicios y prestaciones de sus distintos sistemas sanitarios 3,4. Tal circunstancia hace posible planificar de manera anticipada el modo en que se quiere finalizar con la vida, con independencia de las preferencias del profesional sanitario implicado en este proceso, hecho que limita la actuación clínico-asistencial y evita la distanasia 5,6. No obstante, este derecho, individual e intransferible, queda sujeto a los parámetros de las distintas normativas y la *lex artis ad hoc*^7^.

Las administraciones sanitarias desarrollan programas o procedimientos que permiten establecer una comunicación deliberada y asertiva (entre el profesional sanitario y el paciente) a fin de que se establezcan acuerdos consensuados y consentidos que permitan paliar el sufrimiento de este. Para ello, el paciente puede acogerse al denominado consentimiento informado 8,9. Hacer uso del proceso de la planificación anticipada de las decisiones (PAD) exige conocimiento previo tanto del profesional sanitario como de la persona interesada, además de una actitud favorable a su desarrollo 10,11. Ambos tienen responsabilidad mutua, así como el compromiso de exonerar al otro de la carga emocional que apareja la muerte, sin obviar que la toma de decisiones debe ser un acto compartido 12.

Generar conciencia sobre un tema tan sensible como lo es la muerte digna y sosegada exige el posicionamiento formal de las distintas administraciones públicas. No solo compete a las agencias sanitarias abordar este asunto, sino también a aquellas otras de tipo educativo y social, pues se trata de un derecho multidimensional 13,14. La escuela y la familia son pilares fundamentales para la educación (formal e informal). Estas han de asumir atribuciones afines a la naturalización de la muerte, lejos de mandatos ideológicos, legislativos o constitucionales contrapuestos. Naturalizar el óbito y los tabúes que lo rodean es una cuestión básica, donde la ciudadanía ha de admitir e incorporar a la conciencia colectiva la idea de que la muerte es una etapa más del ciclo vital. Este cambio de prisma debe ir acompañado de novedosas corrientes filosóficas distantes de movimientos conservadores que puedan alentar la culpabilidad y la falsa creencia.

En la actualidad, en los centros educativos apenas existen programas que contemplen materias de semejante calado, con independencia del grado o nivel al que se pertenezca. Los planes docentes y los currículos educativos suelen desestimar esta temática, quedando relegada a una labor pedagógica (transversal) realizada desde el aula a criterio docente, por lo que introducir estos contenidos queda al arbitrio de la susceptibilidad 15. Son necesarias medidas formativas y de sensibilización generadoras del diálogo y la reflexión temprana, donde la ciudadanía alcance a comprender y asumir (sin dramatismo) un desenlace inevitable, pero no por ello menos humanizado. Es ahí donde el derecho a la muerte digna adquiere sentido y forma, y lo hace mediante la PAD y la voluntad vital anticipada 16.

## MÉTODOS

Con este estudio se pretende identificar el conocimiento y la percepción del alumnado universitario sobre el proceso de la muerte digna y su posible difusión ante las agencias sanitarias y la comunidad educativa. Para ello, se realiza un estudio descriptivo transversal en el que participan los estudiantes de posgrado del Máster Universitario en Educación Inclusiva e Intercultural de la Universidad Internacional, de la Universidad Internacional de la Rioja (España), matriculados durante el curso académico 2020-2021. Se lleva a cabo un muestreo aleatorio simple sobre una población total de 480 individuos (IC 95%, ME 5%), cuya N=215. Son criterios de inclusión: tener una edad comprendida entre 25 y 65 años y un claro dominio del castellano. Para este estudio no se contemplan criterios de exclusión.

En cuanto a las variables de estudio, estas se relacionan con el conocimiento y la percepción (como variables dependientes), y el sexo, la edad, la nacionalidad, el nivel de estudios y la profesión/ocupación (como variables independientes).

Con respecto al instrumento para la recolección de datos, se hace uso del cuestionario autoadministrado *ad hoc.* Este se encuentra conformado por preguntas con respuesta de escala numérica y dicotómica, donde la distribución de respuesta oscila entre 0 y 10 o Sí/No/Ns/Nc.

Para la interpretación de los datos, se realiza un análisis descriptivo y bivariante mediante el *software* Epi Info, versión 7.2.

Las limitaciones del estudio se pueden relacionar con la falta de tiempo para participar en el estudio, el insuficiente conocimiento sobre el tema, el conflicto de intereses, el temor a ser cuestionado sobre asuntos relativos al final de la vida y la muerte, entre otras.

## RESULTADOS

En este estudio participaron 136 individuos (tasa de respuesta del 63,2%). La edad media de los participantes es de 37,7 años (rango 24-60; Dt. 8,26). El 66,9% del total son mujeres y el 67,6% tiene nacionalidad colombiana, seguido de aquellos que proceden de Ecuador en un 23,5%. El 86,7% del total cuenta con titulación universitaria (licenciatura); el resto cuenta con otros estudios universitarios. Por su parte, el 39,7% ejerce como profesor de secundaria/ bachillerato, seguido de aquellos que ocupan el puesto de maestro de básica/primaria con un 31,6%.

El conocimiento que estos tienen sobre el derecho a la muerte digna y la normativa que la regula presenta una media de 5,43 (Dt. 2,62) sobre 10. Una media de 6,56 (Dt. 2,61) considera que en su entorno se habla abiertamente y con naturalidad sobre la muerte. Por su parte, una media de 5,34 (Dt. 2,93) manifiesta que hablar sobre el proceso del final de la vida en su entorno es un tema tabú. Centrada la atención en el lugar donde se produce el fallecimiento, una media de 7,22 (Dt. 2,32) entiende que en su comunidad/ciudad los enfermos paliativos (en fase de final de vida) fallecen primordialmente en el ámbito hospitalario. Casi el 34% del total de participantes afirma que el ordenamiento jurídico de su país cuenta con una ley reguladora del derecho a la muerte digna y el 36% se posiciona en la categoría (no sabe/no contesta).

Asimismo, una media de 2,88 (Dt. 2,87) es la respuesta dada a la pregunta relativa al conocimiento de la ciudadanía sobre la existencia de una ley de muerte digna en el país de origen. En el caso en que el país de residencia no contemple (en su marco normativo) una ley de este calibre, una media de 8,26 (Dt. 2,62) considera necesario la aprobación de una ley que permita a la persona decidir a nivel clínico sobre la forma en que desea finalizar con su vida. Este aspecto se relaciona con la actitud que pueda tener la ciudadanía sobre el derecho a la autodeterminación clínica.

No obstante, a la pregunta ¿la ciudadanía tiene una actitud positiva hacia el derecho a decidir los tratamientos clínicos que quiere que se le apliquen en las etapas finales de la vida?, la media de respuesta se sitúa en el 6,35 (Dt. 2,57). En cuanto a determinar a quién le concierne esta decisión, y si esta es compartida entre el profesional sanitario y el paciente, una media de 5,07 (2,88) estima que en su comunidad/ciudad el paciente decide conjuntamente con el médico los tratamientos clínicos que quiere que se le apliquen. Sin embargo, una media de 6,59 (Dt. 2,64) cree que en su comunidad/ciudad la responsabilidad sobre el proceso de salud-enfermedad de los pacientes recae exclusivamente sobre el médico. Respecto de las posibles estrategias desarrolladas por los centros sanitarios garantes de una información adecuada que permita al paciente vincularse a su proceso asistencial, una media de 5,52 (Dt. 2,61) entiende que estos centros implementan estrategias informativas/formativas que involucran al ciudadano en su propio proceso de salud-enfermedad.

Tomada como referencia, los centros escolares y la posibilidad de que estos ofrezcan formación sobre el final de la vida (la muerte) y el derecho a la PAD, el 70,5% de los participantes de este estudio asegura que los centros educativos de su entorno/ciudad no contemplan en el currículo escolar materias específicas que tengan en cuenta dichos derechos. Asimismo, el 80,8% considera que los centros educativos tampoco desarrollan actividades formativas que promuevan el conocimiento del alumnado sobre el derecho a la muerte digna; el 11,7% desconoce tal cuestión. Pese a ello, una media de 9,22 (Dt. 1,66) ve necesario que los colegios ofrezcan información/formación al alumnado sobre el proceso de la vida y la muerte (su significado, sentido, valores, entre otros).

De igual modo, una media de 8,87 (Dt. 1,81) entiende que hablar del proceso de la muerte es un hecho natural y social que debe ser normalizado desde los colegios. Además, consignando aspectos que rodean a la muerte como las creencias religiosas, una media de 6,99 (Dt. 2,84) sostiene que en su comunidad/ciudad las decisiones clínicas (en el proceso del final de la vida) se hallan influidas por los rituales ancestrales y el sistema de valores. Además, una media de 9,26 (Dt. 1,69) es el valor concedido al hecho de consignar que morir con dignidad ha de ser un derecho humano universal (Tablas 1 y 2). Finalmente, respecto del análisis bivariante, cabe decir que no existe relación entre las variables independientes de edad, nivel académico y profesión y el conocimiento que se pueda tener sobre la existencia de leyes reguladoras de la muerte digna (según el país de origen).

**Tabla 1:** Análisis descriptivo de las variables de conocimiento y percepción

Pregunta	Número	Mínimos	Máximos	Media	Varianza	Desviación típica
P1	134,0000	1,0000	10,0000	5,4701	6,9126	2,6292
P2	136,0000	1,0000	10,0000	6,5662	6,8400	2,6153
P3	133,0000	1,0000	10,0000	5,3459	8,5916	2,9311
P4	135,0000	1,0000	10,0000	7,2296	5,4170	2,3275
P5	----	----	----	----	----	----
P6	136,0000	1,0000	NA	2,8897	8,2470	2,8718
P7	123,0000	1,0000	10,0000	8,2683	6,8701	2,6211
P8	135,0000	1,0000	10,0000	6,3556	6,6338	2,5756
P9	134,0000	1,0000	10,0000	5,0746	8,2951	2,8801
P10	135,0000	1,0000	10,0000	6,5926	6,9746	2,6409
P11	134,0000	1,0000	10,0000	5,5299	6,8525	2,6177
P12	----	----	----	----	----	----
P13	----	----	----	----	----	----
P14	135,0000	1,0000	10,0000	9,2222	2,7861	1,6692
P15	136,0000	1,0000	10,0000	8,8750	3,2954	1,8153
P16	135,0000	1,0000	10,0000	6,9926	8,0671	2,8403
P17	136,0000	1,0000	10,0000	9,2647	2,8627	1,6920

P1. Puntúe del 1 al 10 su conocimiento sobre el derecho a la muerte digna y la normativa que lo regula (sepa que 1 equivale a la mínima puntuación o conocimiento y 10 a la máxima puntuación o conocimiento). P2. ¿En su entono se habla abiertamente y con naturalidad sobre la muerte? P3. ¿En su entorno hablar sobre el proceso del final de la vida es un tema tabú?

P4. ¿En su comunidad/ciudad los enfermos paliativos (en fase final de la vida) fallecen principalmente en el hospital? P5. ¿En su país existe una ley reguladora del derecho a la muerte digna? P6. En caso afirmativo ¿La ciudadanía tiene conocimiento de dicha ley?

P7. En caso contrario ¿Considera necesario la aprobación de una ley que permita a la persona decidir sobre las formas en que desea finalizar (clínicamente) con su vida?

P8. ¿La ciudadanía tiene una actitud positiva hacia el derecho a decidir los tratamientos clínicos que quiere que se le apliquen en las etapas finales de la vida?

P9. ¿En su comunidad/ciudad el enfermo decide conjuntamente con el médico los tratamientos clínicos que se le van a aplicar?

P10. ¿En su comunidad/ciudad la responsabilidad sobre el proceso de salud-enfermedad de los ciudadanos/pacientes recae exclusivamente sobre el médico?

P11. Los centros sanitarios de su comunidad/ciudad ¿Desarrollan estrategias informativas/formativas que permitan al ciudadano involucrarse en su propio proceso de salud-enfermedad?

P12. ¿Los centros educativos de su entorno/ciudad contemplan en el currículum materias específicas que tienen en cuenta los derechos sanitarios de los ciudadanos?

P13. ¿Los centros educativos de su comunidad/ciudad desarrollan actividades formativas que promueven el conocimiento de los alumnos acerca del derecho a la muerte digna?

P14. ¿Considera necesario que los colegios ofrezcan información/formación a los alumnos sobre el proceso de la vida y la muerte (su significado, sentido, valores, etc.)?

P15. ¿Hablar del proceso de la muerte es un hecho natural y social que debe ser normalizado desde los colegios?

P16. ¿En su comunidad/ciudad las decisiones clínicas en el proceso del final de la vida están influenciadas por los rituales ancestrales y el sistema de valores?

P17. ¿Morir con dignidad debe ser considerado un derecho humano universal?

**Tabla 2:** Análisis descriptivo de las variables categóricas de conocimiento

	Frecuencia	(%)	(%) acumulado
P5			
NO	42	30,88	30,8
SI	45	33,09	100,00
NS/NC	49	36,03	66,91
Total	136	100,00	100,00
P12			
NO	96	70,59	71,32
SI	29	21,32	100,00
NS/NC	10	7,35	78,68
Total	136	100,00	100,00
P13			
NO	110	80,88	81,62
SI	9	6,62	100,00
NS/NC	16	11,76	93,38
Na	1	0,74	0,74
Total	136	100,00	100,00

P5. ¿En su país existe una ley reguladora del derecho a la muerte digna?

P12. ¿Los centros educativos de su entorno/ciudad contemplan en el currículo materias específicas que tienen en cuenta los derechos sanitarios de los ciudadanos?

P13. ¿Los centros educativos de su comunidad/ciudad desarrollan actividades formativas que promueven el conocimiento de los alumnos acerca del derecho a la muerte digna?

Tampoco existe relación entre variables con la cuestión planteada sobre el papel de los centros educativos para el desarrollo de acciones formativas promotoras del aprendizaje del alumnado sobre el derecho a la muerte digna, así como la implantación de materias específicas en el currículo escolar. No obstante, sí existe relación entre la variable de conocimiento sobre la existencia de leyes reguladoras de la muerte digna y las variables de sexo y nacionalidad (Tablas 3 y 4).

**Tabla 3:** Análisis bivariante de las variables continuas y categóricas del conocimiento de la ley reguladora del derecho a la muerte digna según variables independientes

Pruebas estadísticas				
P5	N	Chi-cuadrado	GL	Probabilidad
Edad	133	13,5582	10	0,1941
Sexo	135	9,474	4	0,0503
Nacionalidad	134	22,3425	10	0,0135
Nivel de estudios	136	4,7339	4	0,3157
Profesión	132	21,9737	16	0,144
P12		Chi-cuadrado	GL	Probabilidad
Edad	133	9,156	15	0,8692
Sexo	135	3,314	6	0,7685
Nacionalidad	134	14,2573	15	0,5061
Nivel de estudios	136	8,6441	6	0,1946
Profesión	132	13,9003	24	0,9489
P13		Chi-cuadrado	GL	Probabilidad
Edad	133	14,6873	15	0,4742
Sexo	135	3,279	6	0,7731
Nacionalidad	134	9,0511	15	0,8748
Nivel de estudios	136	3,1538	6	0,7893
Profesión	132	14,2723	24	0,9403

P5. En su país existe una ley reguladora del derecho a la muerte digna. P12. ¿Los centros educativos de su entorno/ciudad contemplan en el currículo materias específicas que tienen en cuenta los derechos sanitarios de los ciudadanos? P13. ¿Los centros educativos de su comunidad/ciudad desarrollan actividades formativas que promueven el conocimiento de los alumnos acerca del derecho a la muerte digna?

**Tabla 4:** Análisis de las variables continúas del conocimiento según el sexo y la nacionalidad

P5. En su país existe una ley reguladora del derecho a la muerte digna				
	NO (%)	NS/NC (%)	SI (%)	Total (%)
Sexo				
Femenino	25	40	26	91
Row%	27,47	43,96	28,57	100,00
Col%	59,52	81,63	57,78	66,91
Masculino	16	9	19	44
Row%	36,36	20,45	43,18	100,00
Col%	38,10	18,37	42,22	32,35
Na	1	0	0	1
Row%	100,00	0,00	0,00	100,00
Col%	2,38	0,00	0,00	0,74
Total	42	49	45	136
Row%	30,88	36,03	33,09	100,00
Col%	100,00	100,00	100,00	100,00
Nacionalidad	NO	NS/NC	SI	Total
Colombia	28	33	31	92
Row%	30,43	35,87	33,70	100,00
Col%	66,67	67,35	68,89	67,65
Ecuador	11	15	6	32
Row%	97,70	81,61	20,69	100,00
Col%	26,19	30,61	13,33	23,53
España	0	0	7	7
Row%	0,00	0,00	100,00	100,00
Col%	0,00	0,00	15,56	5,15
Na	1	0	1	2
Row%	50,00	0,00	50,00	100,00
Col%	2,38	0,00	2,22	1,47
Panamá	2	1	0	3
Row%	66,67	33,33	0,00	100,00
Col%	4,76	2,04	0,00	2,21
Total	42	49	45	136
Row%	30,88	36,03	33,09	100,00
Col%	100,00	100,00	100,00	100,00

## DISCUSIÓN

El debate sobre el derecho a la muerte digna y su diálogo deliberado son cuestiones controvertidas, cargadas de tabúes y emotividad. Normalizar e integrar dicho debate en los distintos contextos sociopolíticos, económicos, sanitarios, educativos, entre otros, requiere interiorizar el último estadio de la vida como un hecho natural, además de social 17,18.

La aceptación y el raciocinio ciudadano se convierten en ejes centrales para garantizar su fundamento. Tal aceptación precisa conocimiento previo y concienciación por parte de la sociedad. Aquí, los distintos actores/ agentes sanitarios y sociales han de desempeñar una labor de discernimiento crucial que permita el correcto posicionamiento 19. De esta investigación se deduce que el conocimiento que los participantes tienen sobre el derecho a la muerte digna y su normativa es ajustado, por lo que precisan de una mayor formación. Estos consideran que la ciudadanía no cuenta con suficiente información sobre la ley reguladora de la muerte digna, hecho que puede complejizar el desarrollo del documento de voluntades anticipadas y su difusión. Tales datos guardan relación con los resultados del estudio español que C. Santos realizase hace tiempo y quien afirmara que la población adulta tiene un conocimiento insuficiente sobre la voluntad vital anticipada.

Dicho autor abogaba por la necesidad de informar a la ciudadanía sobre este derecho, dado que la actitud de los encuestados era favorable 20. Asimismo, investigaciones más recientes como la elaborada por M.C. Bejarano et al. confirman que la PAD sigue siendo desconocida entre la población española, pues poco más del 50% de las personas a las que encuesta tiene conocimiento de esta 21; circunstancia que podría obstaculizar la muerte digna.

Por su parte, del estudio realizado por J.C. Bermejo et al., en el que participan profesionales de la salud, así como población general con formación universitaria y de bachillerato (principalmente) de diversos puntos de España, se desprende que el 71,8% (sobre una muestra de 370 individuos) sabe de la existencia del proceso de la PAD. Además, al 89,4% le gustaría otorgar su propio documento de voluntades anticipadas; hecho que evita el encarnizamiento terapéutico 22.

No obstante, investigaciones actuales afirman que la PAD sigue siendo un proceso desconocido por la ciudadanía 23, donde influyen variables como la edad, el género, el nivel de instrucción, entre otros, así como su tradición y recorrido histórico según el contexto en el que nos encontremos; conocimiento que se traduce en autodeterminación clínica para el buen morir 24. En otro orden de cosas, de este estudio se deduce que, aun cuando pueda existir cierto grado de tabú a la hora de hablar acerca de la muerte, hay una adecuada predisposición y apertura al discurso ético sobre el derecho a la muerte digna. Máxime, en contextos sanitarios donde se presupone que las decisiones clínicas en los momentos del final de la vida cobran sentido, debiendo ser estas compartidas a fin de reducir la incertidumbre 25,26.

Asimismo, los resultados de este estudio confirman que las decisiones clínicas en la PAD y el proceso de final de la vida están influidas por el sistema de valores de la persona, o al menos por la ritualidad; es decir, la cultura predominante. Respecto de esta cuestión, estudios analíticos como el realizado por la Comisión Autonómica de Ética e Investigación Sanitaria de la Consejería de Salud de la Junta de Andalucía confirman que el proceso de muerte digna está influido por la religiosidad, es decir, la fe y los mandatos eclesiásticos 27.

Por su parte, investigaciones como la realizada por J. M. Jiménez y M. Farouk demuestran que el sistema de creencias y valores de la ciudadanía no es un factor determinante para el inicio de la PAD 28; como tampoco lo es para los profesionales sanitarios, pues entienden que el acompañamiento en el proceso de muerte digna es un deber de la ética profesional 29-31. Sea como fuere, del presente estudio se constata la existencia de una clara conformidad por difundir el derecho a la muerte digna. Se halla unanimidad en los pronunciamientos acerca de la necesidad de formar/informar a la ciudadanía sobre este precepto desde los centros educativos. Centrada la atención sobre la muestra de estudio con nacionalidad en Colombia, esta afirma de forma categórica (en un porcentaje considerable) que en su país no existe una ley reguladora de la muerte digna, o desconoce su existencia.

No obstante, investigaciones como la realizada por A. J. Peña et al., y la propia Corte Constitucional de Colombia, afirman que este derecho se halla incluido en el ordenamiento jurídico colombiano 32. Es más, por mandato expreso de la Sentencia C-239 de 1997, Colombia se convierte en el primer país del mundo en consagrar la muerte digna (ortotanasia) como un derecho fundamental. Esta apreciación denota desconocimiento, como la necesidad de incidir en la formación de la ciudadanía sobre este derecho sanitario.

Para concluir, cabe decir que, pese al conocimiento que se pueda tener respecto del derecho a la muerte digna y el posicionamiento cultural, la percepción favorable sobre este derecho ha de servir como acicate para la acción formativa desde las distintas administraciones públicas. De ahí que las organizaciones sanitarias y educativas deban contribuir al desarrollo de estrategias pedagógicas específicas que garanticen el conocimiento ciudadano y la concienciación de los distintos agentes y actores socio-sanitarios 33. Esto hace posible el fortalecimiento del derecho a la autonomía, el diálogo ético-moral, la toma de decisiones compartidas y la naturalización de la muerte; aspectos que permiten al paciente decidir por sí mismo en los estadios finales de la vida 34 ♦
